# Metagenomic and Metatranscriptomic Analyses of Diverse Watermelon Cultivars Reveal the Role of Fruit Associated Microbiome in Carbohydrate Metabolism and Ripening of Mature Fruits

**DOI:** 10.3389/fpls.2018.00004

**Published:** 2018-01-19

**Authors:** Thangasamy Saminathan, Marleny García, Bandana Ghimire, Carlos Lopez, Abiodun Bodunrin, Padma Nimmakayala, Venkata L. Abburi, Amnon Levi, Nagamani Balagurusamy, Umesh K. Reddy

**Affiliations:** ^1^Gus R. Douglass Institute and Department of Biology, West Virginia State University, Institute, WV, United States; ^2^Laboratorio de Biorremediación, Facultad de Ciencias Biológicas, Universidad Autónoma de Coahuila, Torreón, Mexico; ^3^U.S. Vegetable Laboratory, Agricultural Research Service, United States Department of Agriculture, Charleston, SC, United States

**Keywords:** watermelon, microbiome, ripe fruits, metagenomics, metatranscriptomics

## Abstract

The plant microbiome is a key determinant of plant health and productivity, and changes in the plant microbiome can alter the tolerance to biotic and abiotic stresses and the quality of end produce. Little is known about the microbial diversity and its effect on carbohydrate metabolism in ripe fruits. In this study, we aimed to understand the diversity and function of microorganisms in relation to carbohydrate metabolism of ripe watermelon fruits. We used 16S metagenomics and RNAseq metatranscriptomics for analysis of red (PI459074, Congo, and SDRose) and yellow fruit-flesh cultivars (PI227202, PI435990, and JBush) of geographically and metabolically diverse watermelon cultivars. Metagenomics data showed that Proteobacteria were abundant in SDRose and PI227202, whereas Cyanobacteria were most abundant in Congo and PI4559074. In the case of metatranscriptome data, Proteobacteria was the most abundant in all cultivars. High expression of genes linked to infectious diseases and the expression of peptidoglycan hydrolases associated to pathogenicity of eukaryotic hosts was observed in SDRose, which could have resulted in low microbial diversity in this cultivar. The presence of GH28, associated with polygalacturonase activity in JBush and SDRose could be related to cell wall modifications including de-esterification and depolymerization, and consequent loss of galacturonic acid and neutral sugars. Moreover, based on the KEGG annotation of the expressed genes, nine α-galactosidase genes involved in key processes of galactosyl oligosaccharide metabolism, such as raffinose family were identified and galactose metabolism pathway was reconstructed. Results of this study underline the links between the host and fruit-associated microbiome in carbohydrate metabolism of the ripe fruits. The cultivar difference in watermelon reflects the quantum and diversity of the microbiome, which would benefit watermelon and other plant breeders aiming at the holobiont concept to incorporate associated microbiomes in breeding programs.

## Introduction

Watermelon (*Citrullus lanatus*) is a major cucurbit crop grown in tropical and subtropical regions of the world (Chomicki and Renner, 2015). Because of its nutritional properties, watermelon represents ∼7% of the world area of vegetable cultivation^1^. The watermelon fruit is rich in water (90%), sugar, fiber, vitamins, amino acids, minerals and carotenoids, especially lycopene, flavonoids, and triterpenoids. Nutritional composition of plants is mediated by the different stages of development. A complex and highly coordinated developmental phase of fruit ontogeny is ripe stage, where several physiological changes occur (Gapper et al., 2013). Recently, plants have been considered a holobiont, a unit encompassing both the host and its associated microbiome (Vandenkoornhuyse et al., 2015). The microbiome is associated in the form of colonization outside the plant as well as inside, such as vascular bundles, roots, and leaves (Berendsen et al., 2012). Most microorganisms, particularly those colonizing roots and stems, seem to originate from the rhizosphere and colonize plant organs as part of their life cycle. Some microorganisms are able to move systemically within the plant (Hallmann and Berg, 2006; Rosenblueth and Martínez-Romero, 2006), whereas others are restricted to below-ground parts of plants (Hallman et al., 2001; Compant et al., 2011). This plant-associated microbiome is highly diverse and comprises a range of different taxa (James et al., 1994; Rosenblueth and Martínez-Romero, 2006). Distinct microbial communities in low density have been reported in flowers, seeds, and fruits (Compant et al., 2010).

Interactions between plant tissues and microbiota can be beneficial, including mutualistic interactions that promote plant health and productivity and can have adverse or no effects on the plant phenotype (Müller et al., 2016). The beneficial effect of direct plant growth promotion by microbes is based on improved nutrient acquisition and hormonal stimulation. The presence of neutral and mutualistic microorganisms prevent the colonization of pathogenic microorganisms, thus protecting plants against infectious diseases (Andreote et al., 2014; Vandenkoornhuyse et al., 2015). The reduction in *Fusarium* wilt infection in watermelon has long been observed in soil containing non-pathogenic *Fusarium oxysporum, Pseudomonas fluorescens* and several archaea (Alabouvette and Couteaudier, 1992). One of the mechanisms for disease suppression in plants could be competition for nutrients and colonizing sites (Shimotsuma et al., 1972; Alabouvette and Couteaudier, 1992). However, the taxonomy and metabolism of the plant-associated microbiome can be directly related to the nutrient components present in a specific part of the plant. In addition, microbial community shifts can occur due to environmental factors and plant developmental activity, thereby producing a dynamic process in which the microbial community and the relations between microbe–microbe and microbe–plant (fruit) may strongly vary (Müller et al., 2016).

Traditional studies on plant microbiota have focused on culturable bacterial groups, but they do not give a clear idea of the plant–microbe interactions because of limitations because of unculturable microorganisms. Next-generation sequencing (NGS) technologies have allowed for studying this hidden microbial diversity in terms of different environmental parameters. Several studies have been used NGS to elucidate microbiomes associated with barley (Bulgarelli et al., 2015), corn (Peiffer et al., 2013), lettuce (Rastogi et al., 2012), potato (İnceoğlu et al., 2011), and rice (Edwards et al., 2015) for different developmental aspects. Ripening changes in tomato were found regulated at multiple levels (DNA, RNA, and protein) and dependent on the coordinated activity of multiple plant hormones (Zhong et al., 2013). The modifications during ripe stage include the accumulation of pigments and sugars and the production of aromatic compounds and flesh softening (Gapper et al., 2013). While glucose and fructose are main sugars during the initial phase of watermelon fruit development, sucrose is more than 70% during the ripe stage (Yativ et al., 2010). Textural changes in ripe fruits are highly associated with carbohydrate metabolism. These changes are mainly due to the dissolution of the middle lamella, the reduction of cell-to-cell adhesion and the weakening of parenchyma cell walls as a result of the action of cell wall-modifying enzymes. Pectins, major components of fruit cell walls that contain α-1, 4-linked D-galacturonic acid, are extensively modified in ripe fruits by their involvement in cell wall extension and fruit softening (Jacob et al., 2008). In apple and strawberry, softening was reduced due to downregulation of polygalacturonase genes (Paniagua et al., 2014). In addition, cell wall and middle lamella modifications are accomplished by many ripe stage related genes encoding polygalacturonase, pectin methylesterase, pectate lyase, β-galactosidase, and cellulase (Brummell and Harpster, 2001; Mercado et al., 2011). The endophytes *Bacillus* and *Kocuria* isolated from papaya fruits could produce extracellular enzymes such as amylase, cellulase, pectinase, and xylanase to act on carbohydrate metabolism toward fruit nutrient composition (Krishnan et al., 2012).

Apart from the above-mentioned factors, hormones also regulate ripe stage and pigmentation process. Transcriptional regulation of ripe stage of fruits coincides with the exposition to the growth hormone ethylene (Wechter et al., 2008). It has been reported that watermelon is sensitive to ethylene and under it, this fruit exhibits acute symptoms of softening by the alteration of polygalacturonase, pectinmethylesterase, and α- and β-galactosidase enzymes (Karakurt and Huber, 2002). Ethylene has also been correlated with carotenoid biosynthesis of watermelon at ripe stage (Grassi et al., 2013), and microbial production of ethylene and carotenoids have been reported previously (Tian and Hua, 2010; Jasim et al., 2015).

While, Berendsen et al. (2012) reported that microbiota might play a fundamental role in the regulation of plant development and affect fruit quality and yield. Little information is available on the role of the microbiome in the ripe fruits of watermelon. In this study, we aimed to analyze the microbiome of ripe fruits of watermelon cultivars of yellow and red flesh by employing both 16S metagenomics and metatranscriptomics to understand and predict their role in ripe fruits.

## Materials and Methods

### Plant Material and Growth Conditions

Watermelon fruits from cultivars with red flesh [PI459074, Congo, and Sweet Dakota Rose (SDRose)] and yellow flesh [PI227202, PI435990, and Jubillee Bush (JBush)] were selected based on fruit flesh color. Selfed seeds of selected cultivars were obtained from the germplasm resources information network (GRIN^2^) and were grown in an experimental field at West Virginia State University for two seasons (summer 2015 and 2016). The soil bed was covered with polyethylene mulch and the plants were irrigated daily at regular intervals with a drip system. All agronomic practices including fertilization and insecticide application followed regular agronomic practices.

### Preparation of Fruit-Flesh for DNA and RNA Extraction

Three replications of mature fruits from each cultivar grown in summer 2016 were collected at ripe stage from the field. Ripe fruits were selected based on the following observations: (a) appearance of yellow color of the fruit in the spot touching the ground; (b) the presence of a dried-up stalk attached to the fruit; (c) slightly rough, ridged, and a dull-opaque appearance of rind; and (d) giving a hollow sound when you thump it with your knuckles. Fruit flesh was collected aseptically from all genotypes. The external surface of the watermelon was rinsed with running water, dried and surface-sterilized with 70% ethanol to avoid the interference of epiphytic bacteria contamination. The cutting utensils (knife, spatula) and board were also surface-sterilized with 70% ethanol. Fruits were cut vertically and the middle flesh was scraped out with a sterile spatula. Samples were flash-frozen in liquid nitrogen and stored at -80°C.

### Genomic DNA Isolation

Genomic DNA was isolated from frozen flesh by using a power food-microbial DNA isolation kit (MO BIO Laboratories, United States). An amount of 500 mg fruit flesh was homogenized in phosphate buffer saline solution. The microbial cells were lysed by microbeads with the lysis buffer provided in the kit. DNA quality and quantity were analyzed by use of the Nanodrop spectrophotometer 1100 (Nanodrop, Wilmington, DE, United States). Isolated DNA was stored at -20°C and diluted to 1 ng/μL with sterile water for 16S metagenomic analysis.

### 16S rRNA Library Construction and Sequencing

The 16S rRNA V4 region was amplified with the bacterial primers 515F (5′-GTGCCAGCMGCCGCGGTAA-3′) and 806R (5′-GGACTACHVGGGTWTCTAAT-3′) and archaeal primers U519F (5′-CAGYMGCCRCGGKAAHACC-3′) and 806R (5′-GGACTACHVGGGTWTCTAAT-3′) with a unique barcode. All PCR reactions involved the Phusion High-Fidelity PCR Master Mix (New England Biolabs, United States). Quantification and purification of PCR products involved a standard procedure (Novogene Bioinformatics Technology, Beijing). Sequencing libraries were generated by using the TruSeq DNA PCR-free sample preparation kit (Illumina, United States) as instructed. The library quality was assessed with the Qubit 2.0 Fluorometer (Thermo Scientific) and the library was sequenced on an Illumina HiSeq2500 platform to generate 250 bp paired-end reads.

### Data Analysis of 16S Amplicons

After truncating the barcode and primer sequences, paired-end reads were merged by using FLASH (Magoč and Salzberg, 2011) to obtain raw reads (Supplementary Table S2). Quality filtering on the raw reads involved specific filtering conditions to obtain high-quality clean reads (Bokulich et al., 2013) according to the QIIME quality control process (Caporaso et al., 2010b). The tags were further compared with the reference database (Gold database) by using the UCHIME algorithm (Edgar et al., 2011) to remove chimera sequences and to obtain effective tags. Sequence analysis involved use of Uparse (Edgar, 2013), and sequences with ≥97% similarity were assigned to the same operational taxonomic units (OTUs). A representative sequence for each OTU was screened for species annotation with the GreenGene Database (DeSantis et al., 2006) based on RDP Classifier (Wang et al., 2007). The phylogenetic relationship of different OTUs, differences among dominant species in samples (groups), and multiple sequence alignment were analyzed by using PyNAST v1.2 (Caporaso et al., 2010a) against the “Core Set” dataset in the GreenGene database.

### RNA Extraction for Metatranscriptome Analyses

RNA was extracted from frozen flesh by the TRIzol method (Life Technologies, Carlsbad, CA, United States). Cell lysis involved grinding flesh in liquid nitrogen and further homogenization with TRIzol reagent as suggested by the manufacturer. The extracted total RNA was purified by using the Zymo research purification kit (Zymo Research, Irvine, CA, United States) as described. RNA quality and quantity were analyzed by using agarose gel electrophoresis and the Agilent 2100 Bio-analyzer (Agilent Technologies, Santa Clara, CA, United States); the extracted RNA was stored at -80°C.

### Library Preparation and Sequencing for Metatranscriptome

Ribosomal RNA was removed from total RNA and the mRNA obtained was fragmented randomly in fragmentation buffer before cDNA synthesis. The final cDNA library was ready after purification, terminal repair, A-tailing, ligation of sequencing adapters, size selection and PCR enrichment. The library concentration was quantified by using the Qubit 2.0 fluorometer (Life Technologies, Carlsbad, CA, United States), adjusted to 2 ng/μl before checking the insert size on an Agilent 2100 Bio-analyzer, and quantified to a greater accuracy by quantitative PCR. Finally, the libraries were sequenced with an Illumina HiSeq2500 platform. The Illumina reads for 16S and metatranscriptome were deposited with the Sequence Reads Archive (NBCI) under the following accession numbers (SAMN08118885, SAMN08118886, SAMN08118887, SAMN08118888, SAMN08118889, SAMN08118890, SAMN08118891).

### Removal of Ribosomal RNA Sequences

Raw RNA-Seq reads were first processed to eliminate adapter and low-quality sequences by using the FastQC program^3^. Removal of the rRNA sequences from the dataset involved use of the SortMeRNA software with the default rRNA database included in the software package, which includes 16S, 23S, 18S, and 28S rRNAs (Kopylova et al., 2012; Leimena et al., 2013). The watermelon genome database is a relatively complete one with annotations, ESTs, transcriptome, etc. from cultivars 97103 and Charleston Gray^4^. The sequences obtained in our transcriptomics study were matched against the watermelon database to eliminate all genes that matched the watermelon genome. Blastn was performed on the remaining reads with a minimum alignment bit score of 54 by using a filtering database consisting of complete ribosomal RNA loci and tRNA sequences of bacteria, archaea, and eukaryote taken from the NCBI and SILVA databases (Pruesse et al., 2007). Thus, filtered sequence reads that passed the rRNA/tRNA filter were reconstructed by using Trinity (version r20140413pl); all samples were then integrated before removing redundant ones with CD-HIT-EST (identity threshold set to 0.95) to obtain unigenes.

### Taxonomic Annotation

For taxonomic identity and functional assignment of unigenes, filtered reads were aligned to the NCBI NR database (e-value ≤ 1e-5) by using Blastn. From earlier work, minimum bit score thresholds of 148, 110, and 74 can be used for phylogenetic and functional assignments at genus level (with >80% confidence level), phylogenetic and functional assignment at the family level (with >80% confidence level) and for a reliable function (COG) assignment (with >95% confidence level), respectively (Leimena et al., 2013). The phylogenetic profiling based on mRNA reads at the phylum level involved reads containing minimum bit alignment score of 148 and the highest rank was selected for the species annotation by using the LCA algorithm (applied in MEGAN software system) to ensure its biological significance (Huson et al., 2011). The top 35 phyla in each sample were selected from the results of species annotation and abundance information, and then clustered by their taxonomy information and the inter-sample differences among samples, to obtain a Species Abundance Heat-map.

### Functional Annotation of KEGG, eggNOG, and CAZy

The unigenes were functionally annotated by mapping to different functional protein databases with BLAST software. Because of more than one result for each mapping unigene, the comparison was done to ensure biological significance, and the BLAST Coverage Ratio (BCR) of reference and Query genes were calculated to ensure a BCR (Ref) and BCR (Que) > 40%, then the corresponding functional annotation information was finally summarized for each watermelon cultivar. Predicted unigenes were assigned to COGs (Tatusov et al., 2000) by blast searches against the COG database (NCBI^5^) with e-value < 10^-6^ for COG assignments. The Kyoto Encyclopedia of Genes and Genomes (KEGG) functional annotation (Kanehisa et al., 2008) of identified proteins involved use of the KEGG Automatic Annotation Server (KAAS^6^) (Moriya et al., 2007) based on a bidirectional best hit assignment method.

### Gene Expression and Comparative Analysis

The unigenes were used as a reference to align with RNA-Seq by Expectation-Maximization (RSEM) (Li and Dewey, 2011). Following the alignments, the number of reads mapped to each watermelon cultivar unigene was derived, then normalized to reads per kilobase of exon model per million mapped reads (RPKM). Relative gene expression was determined by counting the number of unigenes assigned to a particular protein-encoding gene. Normalization was obtained by dividing each gene count by the total mRNA read count of each dataset and multiplying by the average of the total mRNA read count across all datasets (Dillies et al., 2013). Metabolic mapping of the metatranscriptome profiles was performed quantitatively by mapping the KEGG annotation of the identified protein sequences into metabolic pathway maps by using the iPath v2 module^7^. Gene expression of the metabolic pathways was indicated by the line width, determined from the log_2_ values of the read count of KEGG-annotated proteins. Reads with alignment bit-scores ≥ 74 were used to create the global metabolic activity pathways.

## Results and Discussion

### Distribution of Bacterial Communities in Watermelon Fruits Based on 16S rRNA Analysis

**FIGURE 1:**
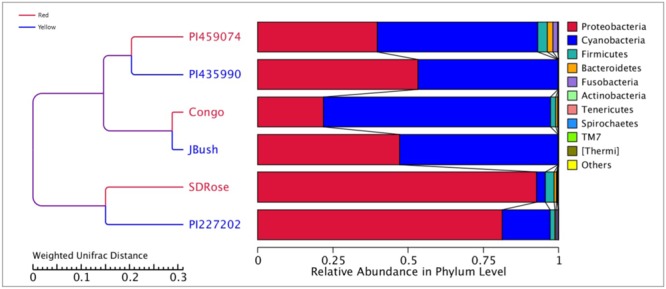
Bacterial diversity at phylum level in ripe fruits of six watermelon cultivars based on 16S rRNA analysis.

We analyzed 16S rRNA to study the bacterial communities associated with red- and yellow-flesh cultivars of watermelon at ripe stage. The cultivar details including total soluble solids and citrulline contents are given in Supplementary Table S1. The red- and yellow-flesh cultivars are from Africa, Asia, and North America. 16S metagenomics sequence data revealed nearly 200,000 raw and clean reads for each cultivar, with average read length of 250 nt (Supplementary Table S2). Proteobacteria was the most abundant phylum in the ripe fruits of watermelon cultivars SDRose, PI227202, and PI435990 (**Figure 1** and Supplementary Table S3). Firmicutes and Bacteroidetes were in less abundance in almost all cultivars tested, and Fusobacteria was recorded highly in PI459074. Proteobacteria represent various taxonomic groups and different ecological statuses, such as endophytes/symbionts (asymptomatic, endophytic bacteria possibly in symbiotic interaction) and saprophytes (bacteria from various environments including soil). Their dominant presence in fruits of watermelon could be attributed to the fruit’s ability to use a wide variety of carbon sources such as carbohydrates, amino acids, and lipids, which could help resist different environmental changes that occur during fruit development (Peighamy-Ashnaei et al., 2006; Kazakov et al., 2009; Xia et al., 2015).

**FIGURE 2:**
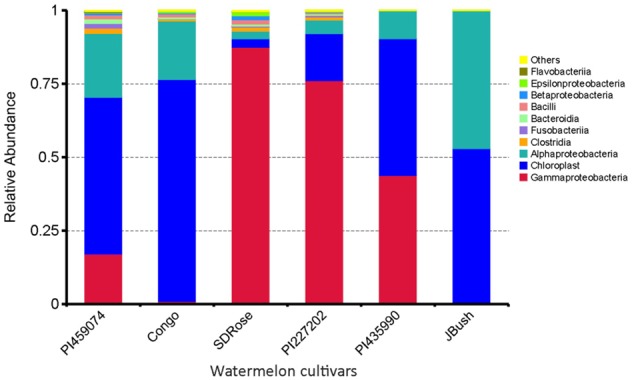
Bacterial community composition by class in ripe fruits of watermelon cultivars based on 16S rRNA analysis.

Earlier, Glassner et al. (2015) reported that Firmicutes, Actinobacteria, β-proteobacteria, and γ-proteobacteria are of major abundance in the flesh of melon fruit, *Cucumis melo* L., another member of the Cucurbitaceae family. Similarly, Proteobacteria, Acidobacteria, Bacteroidetes, and Firmicutes were found the most abundant phyla in grapes (Zarraonaindia et al., 2015). A great percentage of Cyanobacteria was observed in PI459074, Congo, and JBush. They presented a “chloroplast bacterial genome” as a major abundant bacterial class (**Figure 2**). Earlier, plastids were found to have a cyanobacterial ancestor (Douglas and Turner, 1991), and a key role for plastids, specifically chromoplasts, in ripe fruits has been mentioned (Kang et al., 2010). Another important bacterial class in PI459074, Congo and JBush was α-proteobacteria, but in the remaining cultivars, γ-Proteobacteria was the most abundant. The class Bacilli was present in PI459074, Congo, and SDRose; members of this class, such as the *Bacillus* genus, was found predominant in papaya, along with *Kocuria, Acinetobacter*, and *Enterobacter* species (Shi et al., 2010; Krishnan et al., 2012). Antagonistic activity of *Bacillus subtilis* toward fungal and bacterial pathogens of cucurbits is also well-documented (Zeriouh et al., 2014).

**FIGURE 3:**
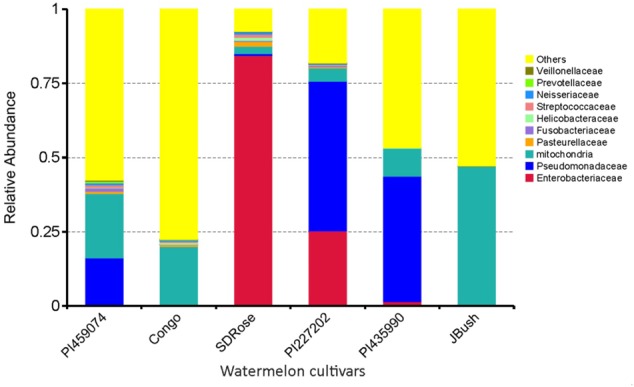
Relative abundance in terms of families of bacteria in ripe fruits of watermelon cultivars based on 16S rRNA analysis.

The relative abundance of bacterial families differed among all tested cultivars, nevertheless very low bacterial diversity was observed in all cultivars. Genes involved in defense response and resistance may undergo differential expression during development and ripe stage of watermelon fruits to control pathogens and consequently could also restrict the establishment of non-pathogenic bacteria, which could explain the reduced bacterial diversity in watermelon fruits (**Figure 3**). Except for SDRose, PI227202 and PI435990, other cultivars showed major abundance among “other” bacterial types. Families belonging to the γ-Proteobacteria class presented the highest abundance. In SDRose, Enterobacteriaceae was the most abundant family (**Figure 3**). Members of Enterobacteriaceae include important pathogens for humans, such as *Salmonella* and *Escherichia coli* O157, even though bacteria belonging to this family were previously isolated from plant tissues, exhibiting antibiotic resistance (Markova et al., 2005). For PI227202 and PI435990, Pseudomonadaceae was the most abundant family. Members of this family have been found as endophytic microorganisms in watermelon. Compant et al. (2011) also observed that *Pseudomonas* was among the predominant bacterial isolates from the interior of flowers, fruits, and seeds of grapevine.

### Active Microbiome Associated with Watermelon Cultivars

**FIGURE 4:**
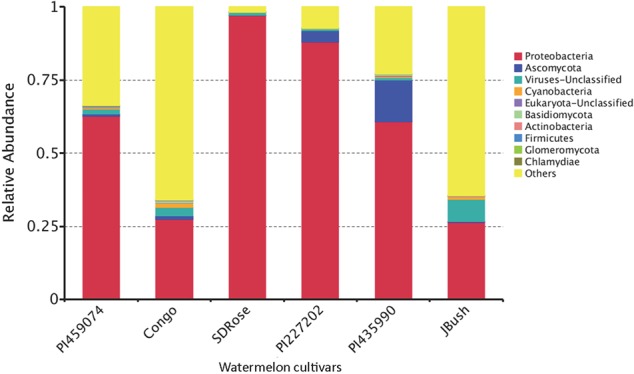
Relative abundance of the transcriptionally active microbiome at phylum level in ripe fruits of watermelon cultivars.

We used metatranscriptomic analysis for in-depth study of the active microbiome and gene expression and associated metabolic pathways in ripe fruits of six cultivars of watermelon fruits. HiSeq2500 generated ∼64, 41, 53, 68, 61, and 33 million paired-end reads for PI459074, Congo, SDRose, PI227202, PI435990, and JBush, respectively. Data on sequenced reads and mapped reads for all six cultivars is in Supplementary Table S4. Bacteria, archaea, fungi, eukaryote, and viruses were found in the ripe fruits of all six watermelon cultivars. Five bacterial phyla (Proteobacteria, Cyanobacteria, Actinobacteria, Firmicutes, and Chlamydiae), three fungal phyla (Ascomycota, Basidiomycota, and Glomeromycota), unclassified eukaryote and unclassified viruses were the top 10 phyla in all samples (**Figure 4**).

Proteobacteria was the most transcriptionally active phyla in all samples. Ascomycota, a fungal group composed of plant and human pathogens and organisms of biotechnological importance was observed along with other fungal phyla, the large fruit-body producer Basidiomycota and the arbuscular mycorrhizal Glomeryomycota (Berbee, 2001). The other bacterial phyla were Firmicutes and Actinobacteria, a well-known secondary metabolites producer, although they were not predominant in all watermelon fruits tested. The obligate intracellular pathogen bacterium Chlamydiae, unclassified eukaryotes, unclassified viruses, and Cyanobacteria showed minor abundance (**Figure 4**). Members of Cyanobacteria have been reported to produce carotenoids, which could contribute to the carotenoid accumulation driven by the plant in the ripe fruits (Lv et al., 2015). Carotenoid production in fruits produce changes in fruit color and also contributes to the biosynthesis of aroma components because carotenoids serve as substrates for the production of norisoprene and monoterpene aroma volatiles of the fruits (Lewinsohn et al., 2005). This capability has been reported in cyanobacterial members such as *Microcystis aeruginosa* (Jüttner, 1976). Apart from their role in carotenoid production, cyanobacteria could also contribute to the aroma and flavor of watermelon fruit. Fungi phyla such as Basidiomycota and Ascomycota present in our findings have been reported to degrade carotenes, resulting in the production of volatile aroma compounds (Zorn et al., 2003).

**FIGURE 5:**
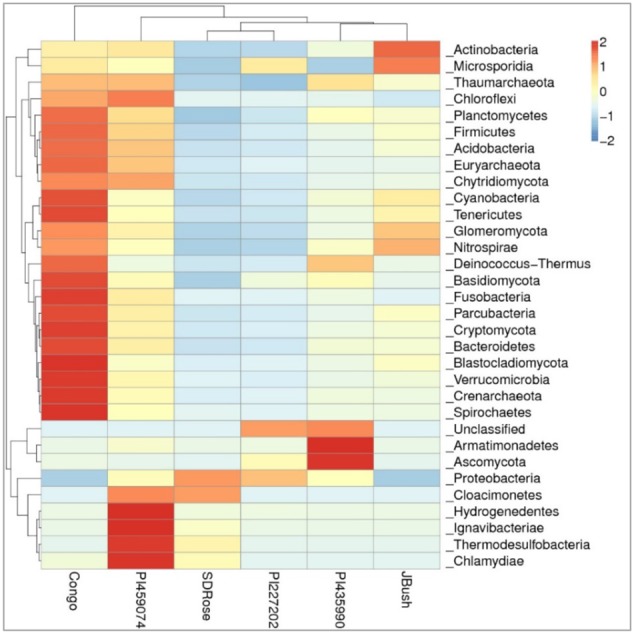
Heatmap of watermelon cultivars based on a comparison of the transcriptionally active microbiome in ripe fruits. Plotted cultivar name on the *X*-axis and selected phyla on the *Y*-axis. The absolute value of “*Z*” represents the distance between the raw score and the population mean in units of the standard deviation. “*Z*” is negative when the raw score is below the mean, positive when above.

In the cultivars PI435990 and PI227202, the most abundant active microbiome was Proteobacteria, Ascomycota, and unclassified viruses. Nevertheless, PI435990 showed Cyanobacteria, unclassified eukaryotes, Basidiomycota, and Chlamydiae in minor abundance. In PI459074 and Congo, Proteobacteria was a dominant phylum, followed by unclassified viruses. Other phyla present in these two varieties were Ascomycota, Actinobacteria, Cyanobacteria, Basidiomycota, and Glomeromycota. SDRose presented less active microbiome diversity, which could be due to the relative predominance of Proteobacteria. Although Proteobacteria were also predominant in JBush, a high abundance of unclassified viruses was also observed, followed by Cyanobacteria, Actinobacteria, and Ascomycota (**Figure 4**). A heatmap is presented to compare the inter-sample differences among the six cultivars of the dominant 35 phyla. Ascomycota and Armatimonadetes phyla showed the highest abundance in the PI435990 cultivar as compared with the other cultivars (**Figure 5**). Armatimonadetes is a recently defined bacterial phylum and is phylogenetically related to Chloroflexi, Actinobacteria, Firmicutes, Deinococcus–Thermus, and Cyanobacteria. Deinococcus–Thermus and Thaumarchaeota, observed in considerably in Congo cultivar, are extremophiles that tolerate oxidation, desiccation, radiation conditions, and biosynthesize carotenoids as a defense mechanism (Tian and Hua, 2010). The presence of Deinococcus–Thermus was reported in apple flower microbiota (Shade et al., 2013) and on the surface of tomato (Telias et al., 2011).

A high abundance of Actinobacteria was observed in the JBush cultivar. Actinobacteria are known for synthesis of secondary metabolites, many of which are found in plants and used as bioactive compounds because of their antimicrobial activity (Fiedler et al., 2008). Furthermore, their diverse metabolism allows them to participate in the metabolism of carbohydrates, including polysaccharides (Pasti and Belli, 1985; Schäfer et al., 1996; Watanabe et al., 2003), which is important in ripe stage of watermelon fruit. The presence of the phylum Glomeromycota in Congo and JBush is significant because the phylum comprises arbuscular mycorrhizal (AM) fungi, which play an important role in plant development and diversity by their phosphate mobilization and nutrient uptake (Bucher, 2007), control of pests and fungal pathogens. The predominance of the Microsporidia phylum in JBush is interesting because it is a known parasite of higher eukaryotes (Keeling and Fast, 2002).

It has been reported in previous studies that the relation between plant-associated microbiome and plant hormones that promote the ripening of fruits (Zouari et al., 2014; Gamalero and Glick, 2015). It is well-known that endogenous and exogenous ethylene triggers in ripe fruits of watermelon through cell wall-degrading enzymes and pectin solubility (Huber et al., 2001; Karakurt and Huber, 2002, 2004). Ethylene forming enzyme 2-oxoglutarate oxygenase/decarboxylase (EFE) of microbial origin produces gaseous ethylene, which subsequently permeates across the bacterial membrane in inducing ripe stage of fruit (Digiacomo et al., 2014). They engineered *E. coli* to synthetize ethylene by the insertion of EFE from *Pseudomonas syringae* to induce the ripe stage in tomato, kiwifruit and apples. In another study, 11 endophytic Proteobacteria belonging to *Pantoea* sp., *Polaromonas* sp., *Pseudomonas* sp., and *Ralstonia* sp. showing 1-aminocyclopropane-1-carboxylate (ACC) deaminase activity were isolated from the fruit tissue of *Elettaria cardamomum* (Jasim et al., 2015). Expression of the ACC deaminase gene has been previously related to ripe tomato (Sheehy et al., 1993). Higher abundance of Proteobacteria described in this present study could play a major role in ripe watermelon fruits.

### Functional Profile of Active Microbiomes of Watermelon Cultivars

The primary cell wall of the fruit contains approximately 35% pectin, 25% cellulose, 20% hemicellulose, and 10% proteins (Brownleader et al., 1999). At ripe stage, the cell wall undergoes different modifications including de-esterification and depolymerization, and consequently loss of galacturonic acid and neutral sugars followed by solubilization of oligosaccharides and remaining sugar residues (Zandleven et al., 2005). An active microbiome could play a major role in this process with its hydrolytic enzymes, in addition to the corresponding enzymes of the host.

**FIGURE 6:**
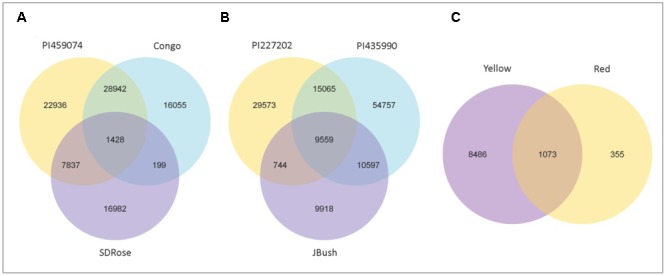
Venn diagram showing the number of shared and unique genes among red types and yellow types and between red and yellow types of watermelon. Gene number among samples of red flesh group **(A)**, yellow flesh group **(B)**, and between red and yellow flesh groups **(C)**. 1428 common genes among red types, 9559 genes were common among yellow types, and 1073 genes were common between yellow and red flesh types.

**FIGURE 7:**
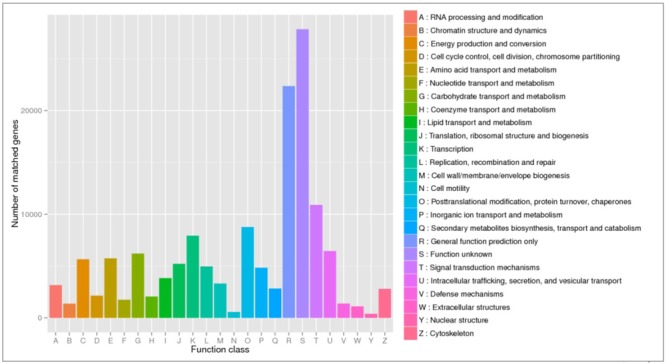
eggNOG functional annotation of orthologous groups among ripe fruits of all watermelon cultivars of this study based on metatranscriptomic data.

**FIGURE 8:**
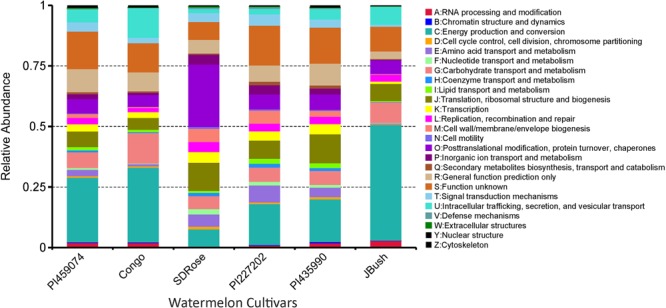
eggNOG function comparison of ripe fruits of watermelon cultivars. Relative abundance of different functional annotation groups was constructed by using metatranscriptome data from the six watermelon cultivars.

**FIGURE 9:**
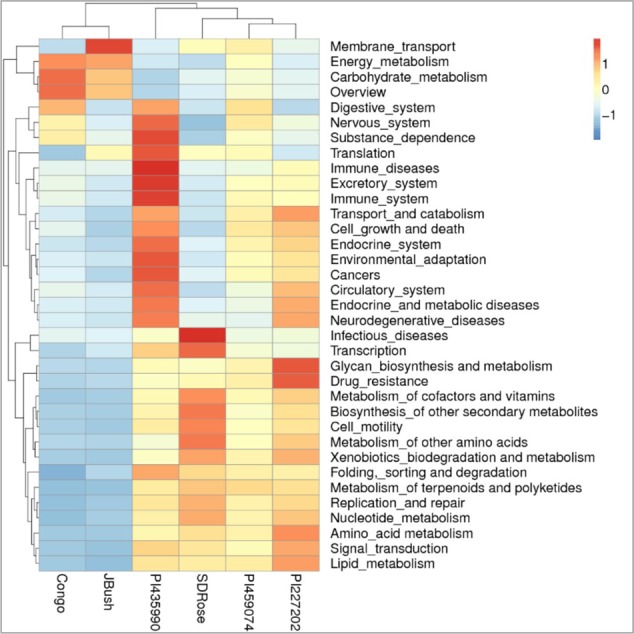
KEGG function comparison of ripe fruits of watermelon cultivars by heatmap analysis. Plotted cultivar name on *X*-axis and KEGG pathways on *Y*-axis. The absolute value of “*Z*” represents the distance between the raw score and the population mean in units of the standard deviation. “*Z*” is negative when the raw score is below the mean, positive when above.

**FIGURE 10:**
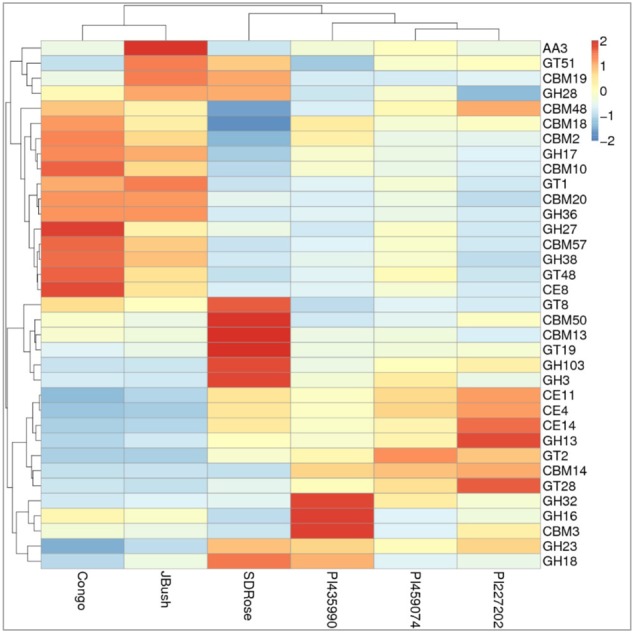
Heatmap of the carbohydrate-active enzymes in watermelon cultivars. Different families of structurally related catalytic and carbohydrate-binding modules of enzymes that degrade, modify, or create glycosidic bonds were found. Within this class of Enzyme Classes were Glycoside hydrolases (GHs), Glycosyltransferases (GTs), Carbohydrate esterases (CEs), Auxiliary activities (AAs), and Carbohydrate-binding modules (CBMs). Plotted cultivar name on *X*-axis and predicted enzymes on *Y*-axis. The absolute value of “*Z*” represents the distance between the raw score and the population mean in units of the standard deviation. “*Z*” is negative when the raw score is below the mean, positive when above.

To obtain general insights into microbial metabolism, transcripts were compared in the following databases: evolutionary genealogy of genes: Non-supervised Orthologous Groups (eggNOG), Kyoto Encyclopedia of Genes and Genomes (KEGG), and Carbohydrate-Active Enzymes Database (CAZy). Based on the analyses of unique and shared genes (**Figure 6**), it was observed that 22,936, 16,055, and 16,982 unique genes were expressed in red flesh cultivars PI459074, Congo, and SDRose, respectively. Similarly, 29,573, 54,757, and 9,918 unique genes were expressed in yellow flesh cultivars PI227202, PI435990, and JBush, respectively. A great sharing of genes was surprisingly observed in yellow and red cultivars, so despite being different cultivars, environmental conditions facilitate the development of relatively similar microbial composition in both types. Genes involved in signal transduction, post-translational modification, transcription, carbohydrate metabolism, intracellular trafficking and amino acid and energy metabolism were the most abundant in all analyzed cultivars (**Figure 7**). The high abundance of these genes could be related to the maintenance of basic cellular machinery, which allowed for growth and cell maintenance of microbial communities during the changes occurring in ripe watermelon. We observed different families of structurally related catalytic and carbohydrate-binding modules (or functional domains) of enzymes that degrade, modify, or create glycosidic bonds. Within this class of enzymes were glycoside hydrolases (GHs), glycosyl transferases (GTs), carbohydrate esterases (CEs), auxiliary activities (AAs), and carbohydrate-binding modules (CBMs) (**Table 1**). Although the microbial diversity of JBush cultivar is low, genes related to energy and carbohydrate metabolism were highly expressed in this cultivar (**Figures 8, 9**). The availability of sugars in ripe watermelon promotes the expression of genes related to carbohydrate metabolism, such as glucosidases, galactosidases, cellulose binding module, and starch binding module (**Figure 10**).

**Table 1:** Carbohydrate-active enzymes (CAZyme) distribution in watermelon varieties.

CAZyme family	Activity	Associated metabolism	Variety
AA3	Integral component of membrane	Membrane transport	JBush
GH3	β-1,4-Glucosidase, β-1,4-xylosidase, β-1,3-glucosidase, β-L-arabinofuranosidase, others	Carbohydrate and energy metabolism	SDRose
GH13	α-Amylase, catalytic domain, and related enzymes	Carbohydrate and energy metabolism	SDRose
GH16	β-1,3(4)-Endoglucanase, others	Energy metabolism	PI435990
GH17	Glucan endo-1,3-β-D-glucosidase glucan 1,3-β-glucosidase, others	Carbohydrate metabolism	Congo
GH18	Chitinase, endo-β-*N*-acetylglucosaminidase, non-catalytic Proteins	Aminoacid metabolism	SDRose
GH23	G-Type lysozyme, peptidoglycan lytic transglycosylase	Membrane transport	SDRose
GH27	α-Galactosidase, α-*N*-acetylgalactosaminidase, Isomalto-dextranase	Carbohydrate and energy metabolism	Congo
GH28	Polygalacturonase, rhamnogalacturonase Others	Carbohydrate metabolism	JBush SDRose
GH32	Invertase, others	Carbohydrate metabolism	PI435990
GH36	α-Galactosidase, α-*N*-acetylgalactosaminidase	Carbohydrate metabolism	Congo JBush
GH103	Peptidoglycan lytic transglycosylase	Membrane transport	SDRose
GT1	UDP-Glucuronosyltransferase 1-β-Galactosyltransferase	Carbohydrate metabolism	JBush
GT2	Cellulose synthase Chitin synthase	Carbohydrate metabolism	PI459074
GT8	Lipopolysaccharide α-1,3-galactosyltransferase	Lipid metabolism	SDRose
GT19	Lipid-A-disaccharide synthase	Membrane transport	SDRose
GT28	1,2-Diacylglycerol 3-β-Galactosyltransferase	Carbohydrate metabolism	PI227202
GT48	1,3-β-Glucan synthase	Carbohydrate metabolism	Congo
GT51	Murein polymerase	Membrane support	JBush
CE4	Acetyl xylan esterase Chitin deacetylase	Carbohydrate metabolism	PI227202
CE8	Pectin methylesterase	Carbohydrate metabolism	Congo
CE11	UDP-3-0-Acyl-*N*-acetylglucosamine deacetylase	Carbohydrate metabolism	PI227202
CE14	*N*-Acetyl -1-D-myo-inosityl-2-amino-2-deoxy-α-D-glucopyranoside deacetylase	Carbohydrate metabolism	PI227202
CBM2	Cellulose-binding domain	Carbohydrate metabolism	Congo
CBM3	Cellulose-binding domain	Carbohydrate metabolism	PI435990
CBM10	Cellulose-binding domain (aerobic bacteria) and dockerin (anaerobic fungi)	Carbohydrate and energy metabolism	Congo
CBM13	Mannose- and xylan-binding domain	Carbohydrate metabolism	SDRose
CBM14	Chitin-binding domain	Structure and Carbohydrate metabolism	PI227202
CBM18	Chitin-binding domain (eukaryotic only)	Structure and Carbohydrate metabolism	Congo
CBM19	Chitin-binding domain (eukaryotic only)	Structure and Carbohydrate metabolism	JBush
CBM20	Starch-binding domain	Carbohydrate metabolism	Congo JBush
CBM48	Glycogen-binding domain	Carbohydrate metabolism	PI227202
CBM50	Peptidoglycan metabolic process	Membrane metabolism	SDRose
CBM57	Quinoprotein amine dehydrogenase, β-chain-like	Signal transduction	Congo

However, in the case of SDRose, despite low expression of genes related to energy metabolism (<10%), high expression of post-translational modification genes (∼25%) was observed (**Figure 8**). In addition, the expression of genes related to infectious diseases was observed (**Figure 9**), which could explain the low microbiome diversity expressed in this cultivar. Many bacterial genera of the most abundant Proteobacteria phylum in this cultivar, such as *Xanthomonas, Erwinia* and *Pseudomonas*, are known pathogens (Kersters et al., 2006). Moreover, fruits at ripe stage are more vulnerable to pathogen attack and environmental stress (Levi et al., 2006). Among the different enzymes expressed in this cultivar, peptidoglycan hydrolases were present. Bacteria use this enzyme for cell wall assembly and disassembly during growth and division; nonetheless, pathogenic bacterial species also use them to cause pathogenicity in eukaryotic hosts (Humann and Lenz, 2009).

On eggNOG annotation, PI227202 and PI435990 cultivars showed a similar pattern of gene expression (**Figure 8**). Regardless, these cultivars presented differences in the expression of genes encoding the CAZ. GH32, associated with hydrolysis of fructans; GH16, associated with the hydrolysis of galactose-containing polysaccharides and galactose monomers; and CBM3, linked with cellulose and chitin binding were observed in major abundance in PI435990 as compared with the other cultivars (**Figure 10**). CEs (CE11, CE4, CE14), associated with polysaccharide deacetylation, glycosyltransferase protein-associated GT28, amylase activity-associated GH13 and glycogen-binding protein-associated CBM48 were predominant in Pl227202. These results suggest the role of microbiome-associated gene expression of carbohydrate metabolism in ripe watermelon fruits.

As mentioned above, cell wall modifications include de-esterification and depolymerization, and consequent loss of galacturonic acid and neutral sugars followed by solubilization of oligosaccharides and remaining sugar residues. The presence of GH28, associated with polygalacturonase activity in JBush and SDRose could be related to cell wall modifications (Zandleven et al., 2005). Further, alkaline pectinases also have been correlated in softening of ripe fruits. *Bacillus*, belonging to Firmicutes (Kapoor et al., 2001) *Pseudomonas* sp. of Proteobacteria (Hayashi et al., 1997) and actinomycetes (Beg et al., 2000a,b) have been reported for their alkaline pectinase activity.

### Predicted Pathways of Carbohydrate Metabolism

**FIGURE 11:**
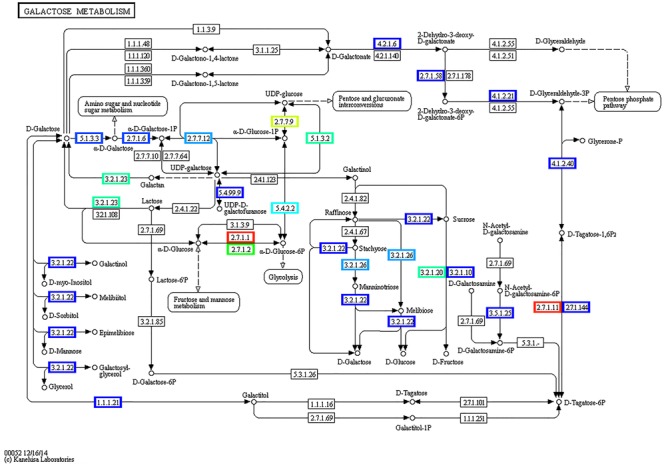
Reconstructed pathway of galactose metabolism based on metatranscriptomic data by KEGG annotation. Predicted enzymes from metatranscriptomic data were associated with galactose metabolism, displayed in this figure.

KEGG annotation of the expressed genes revealed nine α-galactosidase genes involved in key processes of galactosyl oligosaccharide metabolism, such as genes belonged to raffinose family oligosaccharides. Based on these genes, the pathway of galactose metabolism was reconstructed (**Figure 11**). In watermelon fruits, the main free sugars in tissues are sucrose and hexoses (Yativ et al., 2010). In the pathway of sucrose formation, stachyose is converted into sucrose via a sequential action of various enzymes (Dai et al., 2006; Yativ et al., 2010) such as α-galactosidase, which converts stachyose to raffinose and galactose, followed by galactokinase conversion of galactose to galactose-1-phosphate, which is converted to UDP-galactose by UDP-galactose pyrophosphorylase. Later, UDP-galactose-4-epimerase acts on UDP-galactose to form UDP glucose, which is converted to sucrose by sucrose synthase activity combined with fructose. Reconstruction of the galactose metabolism pathway based on our results clearly demonstrated the role of the active microbiome and the gene expression of carbohydrate-active enzymes of ripe watermelon fruits.

Results of this study showed the presence of different phyla of microbiome in ripe watermelon suggested the important role of different classes of bacteria in ripe stage through their metabolic activity and gene expression in carbohydrate metabolism. Moreover, it can be assumed that new phenotypes, without altering the plant genomic information can be developed because of the dynamic interactions between plants and their associated microbiome. The role of plant-associated microbiome in hormonal control during different developmental stages of fruits is a meaningful aspect to understand and requires further studies.

## Author Contributions

UR, PN, TS, and NB designed the plan of study. VA and AL maintained the research materials and recorded the phenotypic data. TS, BG, and AB extracted genomic DNA and RNA from watermelon fruit flesh. NB, UR, TS, MG, and CL analyzed the data using the bioinformatic pipeline. TS, MG, BG, CL, PN, NB, and UR wrote and approved the final version of the manuscript. All authors agree to account for the accuracy and integrity of the paper.

## Conflict of Interest Statement

The authors declare that the research was conducted in the absence of any commercial or financial relationships that could be construed as a potential conflict of interest.

## Footnotes

1 http://www.fao.org/faostat

2 https://www.ars-grin.gov/

3 http://www.bioinformatics.babraham.ac.uk/projects/fastqc/

4 http://cucurbitgenomics.org/

5 ftp://ftp.ncbi.nih.gov/pub/COG/COG

6 http://www.genome.jp/tools/kaas/

7 https://pathways.embl.de/
