# Supplementary material for: Metagenomic and Metatranscriptomic Analyses of Diverse Watermelon Cultivars Reveal the Role of Fruit Associated Microbiome in Carbohydrate Metabolism and Ripening of Mature Fruits
Source: Front Plant Sci. 2018 Jan 19;9:4. doi: 10.3389/fpls.2018.00004 (PMC5780703; doi:10.3389/fpls.2018.00004)
Supplement: Supplementary file 1 [file Data_Sheet_1.pdf]

Table S1. Origin and characteristics of watermelon cultivars.

| Cultivar | Country | Flesh color | Citrulline (mg/g of dry weight) | TSS (%) | Shape/seed number |
|---|---|---|---|---|---|
| PI459074 | Botswana | Red | 11.62 | 6.70 | Oblong/few |
| Congo | Kenya | Red | 10.82 | 10.98 | Oblong/moderate |
| PI227202 | Japan | Yellow | 10.85 | 6.80 | Round/many |
| PI435990 | China | Yellow | 5.79 | 10.50 | Oval/many |
| Jubilee Bush | USA | Yellow | 12.66 | 8.09 | Oval/moderate |
| SD Rose | USA | Red | 16.23 | 10.36 | Round/few |

TSS; Total Soluble Solids.

Table S2. Sequence statistics of 16S metagenomics sequence from watermelon cultivars.

| Sample Name | Raw reads | Clean reads | No chimeras | Avg. Length (nt) | Q20 | Q30 | GC% | Effective% |
|---|---|---|---|---|---|---|---|---|
| Congo | 205,232 | 203,845 | 201,811 | 253 | 99.5 | 98.9 | 55.26 | 98.33 |
| JBush | 208,029 | 206,680 | 204,884 | 252 | 99.5 | 99.0 | 55.53 | 98.49 |
| PI227202 | 211,437 | 210,223 | 205,165 | 253 | 99.6 | 99.1 | 53.41 | 97.03 |
| PI435990 | 216,984 | 215,930 | 213,741 | 253 | 99.6 | 99.1 | 53.53 | 98.51 |
| PI459074 | 218,930 | 217,466 | 214,700 | 252 | 99.5 | 98.9 | 54.36 | 98.07 |
| SDRose | 202,524 | 200,801 | 198,136 | 253 | 99.5 | 99.0 | 56.49 | 97.83 |

Table S3. Values for bacterial diversity in watermelon cultivars at phylum level.

| Taxonomy | PI459074 | Congo | SDRose | PI227202 | PI435990 | JBush |
|---|---|---|---|---|---|---|
| Proteobacteria | 84954 | 43888 | 183136 | 166223 | 113787 | 96408 |
| Cyanobacteria | 114080 | 152047 | 5708 | 32731 | 99413 | 107799 |
| Firmicutes | 6890 | 3366 | 5800 | 3161 | 220 | 119 |
| Bacteroidetes | 3839 | 1471 | 1448 | 966 | 25 | 30 |
| Fusobacteria | 3469 | 179 | 632 | 1191 | 15 | 8 |
| Actinobacteria | 171 | 109 | 298 | 77 | 1 | 9 |
| Tenericutes | 128 | 51 | 112 | 88 | 0 | 1 |
| Spirochaetes | 93 | 38 | 106 | 61 | 0 | 2 |
| TM7 | 41 | 20 | 25 | 5 | 0 | 1 |
| Thermi | 1 | 0 | 22 | 0 | 0 | 0 |
| Verrucomicrobia | 11 | 10 | 15 | 10 | 0 | 0 |
| Acidobacteria | 7 | 3 | 14 | 5 | 0 | 0 |
| SR1 | 8 | 1 | 6 | 1 | 0 | 0 |
| Gemmatimonadetes | 3 | 2 | 7 | 4 | 0 | 0 |
| GN02 | 3 | 1 | 6 | 0 | 0 | 0 |
| Synergistetes | 6 | 2 | 3 | 2 | 0 | 0 |
| Deferribacteres | 2 | 2 | 5 | 1 | 1 | 0 |
| Chloroflexi | 1 | 2 | 3 | 1 | 0 | 0 |
| Fibrobacteres | 1 | 0 | 2 | 0 | 0 | 0 |
| Elusimicrobia | 0 | 0 | 2 | 0 | 0 | 0 |
| WS3 | 1 | 1 | 0 | 0 | 0 | 0 |
| Others | 131 | 13 | 90 | 87 | 2 | 2 |

Table S4. Sequence statistics of metatranscriptomic data of watermelon accessions.

| Sample | Raw Reads | Clean Reads | Clean Bases | Error (%) | Q20 (%) | Q30 (%) | GC Content (%) |
|---|---|---|---|---|---|---|---|
| PI459074 | 73423382 | 64289148 | 9.64G | 0.01 | 98.32 | 95.87 | 49.77 |
| Congo | 62328402 | 41220428 | 6.18G | 0.02 | 96.75 | 92.19 | 47.91 |
| SDRose | 64154274 | 53318946 | 8.00G | 0.01 | 97.84 | 94.88 | 54.00 |
| PI227202 | 73347818 | 68188988 | 10.23G | 0.01 | 98.30 | 95.58 | 52.38 |
| PI435990 | 70411320 | 61069642 | 9.16G | 0.01 | 98.28 | 95.58 | 50.01 |
| JBush | 41017876 | 33241816 | 4.99G | 0.02 | 96.50 | 91.79 | 48.12 |
